# QTc prolongation after aneurysmal subarachnoid hemorrhage might be associated with worse neurologic outcome in patients receiving microsurgical clipping or embolization of the intracranial aneurysms: a retrospective observational study

**DOI:** 10.1186/s12883-024-03679-z

**Published:** 2024-05-23

**Authors:** Xinmin Zhang, Yang Lei, Ling Nan, Su Dong, Yadong Liu, Jinlu Yu, Kan Xu, Kun Hou, Haichun Ma

**Affiliations:** 1 Department of Anesthesiology, First Hospital of Jilin University, 1 Xinmin Avenue, Changchun, 130021 China; 2 Department of Anesthesiology, Liaoyuan Hospital of Traditional Chinese Medicine, Liaoyuan, China; 3 Department of Neurosurgery, First Hospital of Jilin University, 1 Xinmin Avenue, Changchun, 130021 China

**Keywords:** Aneurysmal subarachnoid hemorrhage, Corrected QT interval prolongation, Embolization, Intracranial aneurysm, Microsurgical clipping, Propensity score matching

## Abstract

**Purpose:**

QT interval prolongation is one of the most common electrocardiographic (ECG) abnormalities in patients with aneurysmal subarachnoid hemorrhage (aSAH). Whether corrected QT interval (QTc) prolongation is associated with perioperative cardiac events and dismal neurological outcome in mid to long-term follow-up in patients after aSAH is insufficiently studied and remains controversial.

**Methods:**

We retrospectively studied the adult (≥ 18 years) patients admitted to our institution between Jan 2018 and Dec 2020 for aSAH who underwent intracranial aneurysm clipping or embolization. The patients were divided into 2 groups (normal and QTc prolongation groups) according to their QTc. To minimize the confounding bias, a propensity score matching (PSM) analysis was performed to compare the neurologic outcomes between patients with normal QTc and QTc prolongation.

**Results:**

After screening, 908 patients were finally included. The patients were divided into 2 groups: normal QTc groups (*n* = 714) and long QTc group (*n* = 194). Female sex, hypokalemia, posterior circulation aneurysm, and higher Hunt-Hess grade were associated with QTc prolongation. In multiple regression analysis, older age, higher hemoglobin level, posterior circulation aneurysm, and higher Hunt-Hess grade were identified to be associated with worse outcome during 1-year follow-up. Before PSM, patients with QTc prolongation had higher rate of perioperative cardiac arrest or ventricular arrhythmias. After PSM, there was no statistical difference between normal and QTc prolongation groups in perioperative cardiac events. However, patients in the QTc prolongation group still had worse neurologic outcome during 1-year follow-up.

**Conclusions:**

QTc prolongation is associated with worse outcome in patients following SAH, which is independent of perioperative cardiac events.

## Introduction

QT interval prolongation is one of the most common electrocardiographic (ECG) abnormalities in patients with aneurysmal subarachnoid hemorrhage (aSAH) [1–3]. The normal QT interval varies inversely with heart rate, thus the corrected QT interval (QTc) was popularly used [4]. It has been widely accepted that prolonged QTc is associated with life-threatening ventricular arrhythmias (VAs), such as torsades de pointes (Tdp), and a potentially dismal outcome [5]. It has been reported that more than 40% of patients could experience serious cardiac arrhythmias after aSAH and serious VAs were associated with QTc prolongation and hypokalemia [6]. According to Pasquale et al.’s study, the incidence of QTc prolongation and TdP were 43% and 6% in the acute stage after aSAH, respectively [7]. Hence, aSAH is strongly associated with QTc prolongation and serious cardiac arrhythmias.

Patients with QTc abnormities are at higher risk of intra-operative malignant ventricular arrhythmias after anesthesia [8–11]. It had been reported that QTc prolongation (≥ 480 ms) was associated with a five-fold increase in the risk of cardiac arrest and VAs in liver transplantation [12]. Bradycardia, relative tachycardia, and non-specific ST and T wave abnormalities are strongly and independently associated with 3-month mortality after aSAH [13]. Whether QTc prolongation is associated with cardiac and non-cardiac morbidity and mortality and dismal neurological outcome after aSAH is insufficiently studied and remains controversial [13–15].

In this study we aim to assess the relationship between QTc prolongation and neurological outcome after aSAH.

## Methods

### Study population

We retrospectively studied the adult (≥ 18 years) patients admitted to our institution between Jan 2018 and Dec 2020 for aSAH who underwent intracranial aneurysm clipping or embolization. This study was approved by the institutional ethics committee.

The patients were divided into 2 groups according to their QTc. The extracted clinical data included age, sex, electrocardiographic parameters, biochemistry, cardiovascular risk factors, QT-prolonging medication use, location of aneurysm, Hunt-Hess grade, cardiac arrest or ventricular arrhythmias events, mortality, modified Rankin Scale score (mRS) at discharge and the last follow-up. An mRS score of ≤ 2 was deemed as good outcome. And an mRS score of > 2 was deemed as worse neurological outcome.

Exclusion criteria were:

(1) heart diseases (Coronary heart disease, valvular heart disease, cardiomyopathy); (2) previous neurological diseases or intracranial injury; (3) QTc prolonging drugs; (4) congenital long QTc syndrome; (5) cardiac pacing; (6) complete bundle branch blocks; (7) severe hepatic dysfunction or drug-drug interactions; (8) Patients without ECG; (9) II or III degree atrioventricular block.

### ECG analysis

The QT intervals were corrected for heart rate with the Bazett formula: QTc = QT/square root of RR interval. Patients whose QTc ≥ 470 ms in men and ≥ 480 ms in women belonged to the QTc prolongation group, QTc < 470 ms in men and < 480 ms in women belonged to Normal QTc group [16].

### Statistical analysis

Statistical analysis was performed using the SPSS (version 25.0). Continuous variables were expressed as mean ± standard deviation. Comparison between continuous variables were conducted using the Student’s t-test or Mann-Whitney U test and that for categorical variables were conducted using the Pearson’s chi-square test or Fisher’s exact test. *P* < 0.05 was considered with statistical difference. In univariate analysis, we compared demographic characteristics, past medical history, ECG characteristics, and laboratory values between subjects with normal QTc and QTc prolongation. Variables with statistical difference (*P* ≤ 0.05) during univariate analysis were included in multivariate logistic regression analysis. To minimize the confounding bias, a propensity score matching (PSM) analysis was performed to compare the clinical outcomes between patients with normal QTc and QTc prolongation. Age, hemoglobin level, hypertension, location of aneurysm, and Hunt-Hess grade were selected to create a propensity score ranging from 0 to 1 using logistic regression model. The matching rate was 1:1 and the caliper width was set at 0.2. Each case involving normal QTc was matched to a case of QTc prolongation with the nearest propensity score.

## Results

### QTc interval and perioperative indicators in aSAH patients

**Fig. 1:**
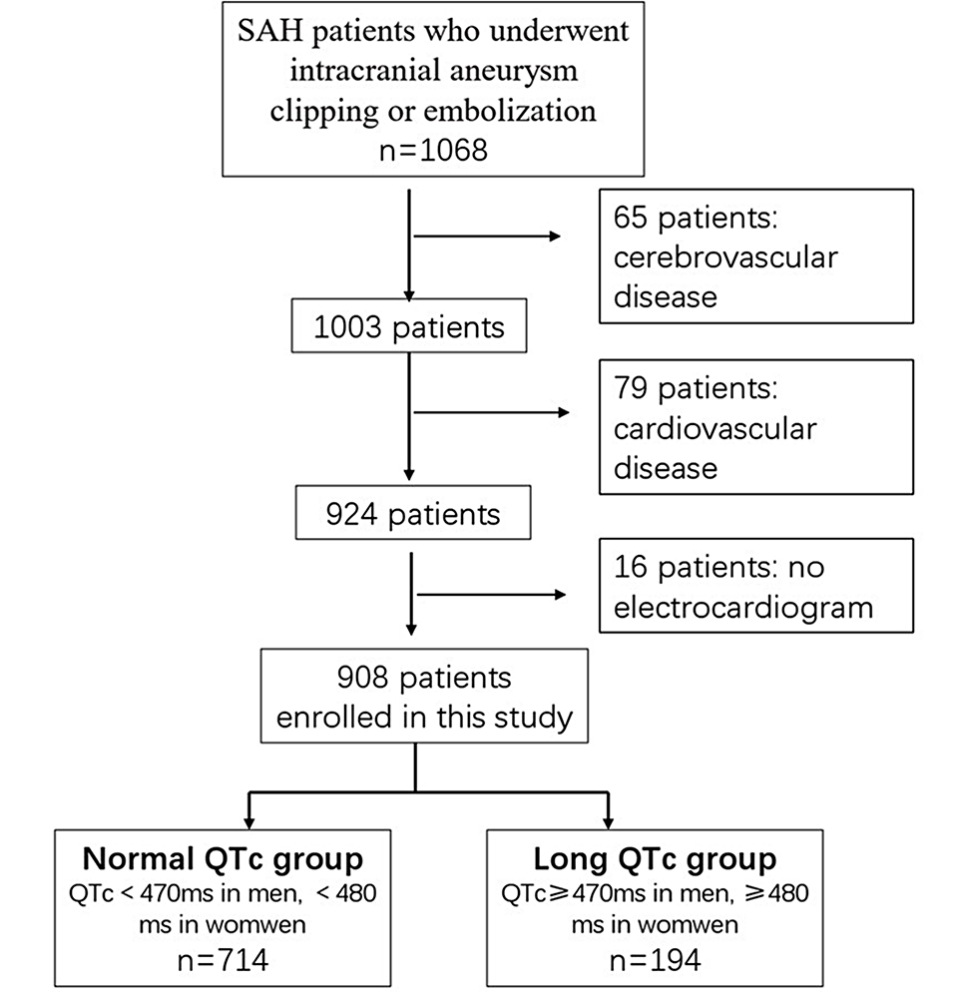
Flow-chart of patients screening

Initially, we identified 1068 patients who underwent intracranial aneurysm clipping or embolization. After screening, 908 patients were finally included (Fig. 1). The patients were divided into 2 groups: normal QTc groups (*n* = 714) and QTc prolongation group (*n* = 194). The QTc was divided with a cutoff value of 470 ms in men and 480 ms in women.

To identify determinants of QTc prolongation, correlation with SAH severity, hemodynamic and biochemical markers and factors that may affect QTc length were assessed. The baseline characteristics and risk factors for QTc prolongation are summarized in Table 1.

**Table 1:** Statistical analysis of various factors for predicting QTc prolongation in patients after aSAH

	Normal QTc (*n* = 714)	QTc prolongation (*n* = 194)	Univariate analysis	Multivariate logistic regression analysis	
			*P* value	*P* value	OR (95% CI)
Age (years), mean ± SD	56.0 ± 9.8	58.2 ± 9.8	0.003	0.062	1.017 (0.999–1.035)
Sex (Male)	268 (37.5%)	46 (23.7%)	<0.001	0.007	0.592(0.404–0.868)
Hb, mean ± SD	136.2 ± 17.4	136.0 ± 18.8	0.967		
Hypokalemia (<3.5mmol/L)	158 (22.1%)	73 (37.6%)	<0.001	<0.001	0.459 (0.314–0.671)
Ca^2+^, mean ± SD	2.25 ± 0.15	2.22 ± 0.13	0.05		
Hypertension	356 (49.9%)	114 (58.8%)	0.028	0.518	0.892 (0.631–1.261)
Diabetes	34 (4.8%)	16 (8.2%)	0.059		
Smoking	266 (37.3%)	73 (37.6%)	0.924		
Location of aneurysm, n (%)			0.001	0.005	2.251 (1.277–3.967)
Anterior circulation	673 (94.3%)	170 (87.6%)			
Posterior circulation	41 (5.7%)	24 (12.4%)			
Hunt-Hess grade			<0.001	0.007	1.771 (1.172–2.675)
I-III	691 (96.8%)	172 (88.7%)			
IV	23 (3.2%)	22 (11.3%)			

Abbreviations: aSAH, aneurysmal subarachnoid hemorrhage; Hb, hemoglobin; QTc, corrected QT interval; SD, standard deviation

### Factors potentially associated with QTc prolongation in SAH patients

To explore the factors associated with QTc prolongation in SAH patients, univariate and multivariate regression analyses were carried out. In univariate analysis, according to electrocardiographic parameters, the patients in long QTc group were prone be older (58.2 ± 9.8 vs. 56.0 ± 9.8, *p* = 0.003) with higher Hunt-Hess grade IV (11.3% vs. 3.2%, *p*<0.001 ) and higher rate of hypertension (58.8% vs. 49.9%, *p*<0.05). The long QTc patients were prone to have hypokalemia (37.6% vs. 22.1%, *p*<0.001) and posterior circulation aneurysm (12.4% vs. 5.7%, *p* = 0.001). Less male gender patients had QTc prolongation (23.7% vs. 37.5%, *p*<0.001). In addition, patients with longer QTc interval had higher cardiac arrest or ventricular arrhythmias in perioperative period ( 3.6% vs. o.7%, *p* = 0.005 ). After multivariate logistic regression analysis, sex, hypokalemia, aneurysm location, and Hunt-Hess grade were still with statistical significance (Table 1).

### Risk factors for worse outcome (mRS > 2)at 1 year follow-up

To identify risk factors for worse outcome ( mRS > 2) at 1 year follow-up, we did univariate analysis firstly, the worse outcome patients were with higher age (60 ± 14 vs. 56 ± 14, *p* = 0.001 ), higher hemoglobin level (139.0 ± 18.2 vs. 135.4 ± 17.5, *p* = 0.022) and longer QTc 【457 (492 − 433) vs. 444 (469 − 424), *p*<0.001】. In addition, hypertension, posterior circulation aneurysm, and higher Hunt-Hess grade were associated with worse outcome. After multivariate logistic regression analysis, only older age, higher hemoglobin level, posterior circulation aneurysm, and higher Hunt-Hess grade were still with statistical significance (Table 2).

**Table 2:** Risk factors for worse outcome(mRS > 2)at 1-year follow-up

			Univariate analysis	Multivariate logistic regression analysis	
	Good outcome(*n* = 733)	Worse outcome(*n* = 175)	P value	P value	OR (95% CI)
Age (years), mean ± SD	56 ± 14	60 ± 14	0.001	0.037	1.022 (1.001–1.042)
Sex, (Male)	248 (33.8%)	66 (37.7%)	0.332		
Hb, mean ± SD	135.4 ± 17.5	139.0 ± 18.2	0.022	0.206	1.007 (0.996–1.019)
Hypokalemia(<3.5mmol/L)	179 (24.4%)	52 (29.7%)	0.425		
Ca^2+^, mean ± SD	2.2 ± 0.1	2.2 ± 0.1	0.892		
QTc	444 (469 − 424)	457 (492 − 433)	<0.001	0.097	1.015 (0.999–1.007)
Hypertension	364 (49.7%)	105 (60%)	0.015	0.303	0.811 (0.544–1.208)
Diabetes	40 (5.5%)	10 (5.7%)	0.893		
Smoking	276 (37.7%)	63 (36.0%)	0.685		
Location of aneurysm, n (%)			0.001	0.003	2.670 (1.406–5.072)
Anterior circulation	691 (94.3%)	152 (86.9%)			
Posterior circulation	42 (5.7%)	23 (13.1%)			
Hunt-Hess grade			<0.001	<0.001	10.621 (7.021–16.066)
I-III	725 (98.9%)	138 (78.9%)			
IV	8 (1.1%)	37 (21.1%)			

Abbreviations: Hb, hemoglobin; mRS, modified Rankin Scale; QTc, corrected QT interval; SD, standard deviation

### Perioperative cardiac events and neurologic outcomes at 1 year follow-up before and after PSM

Age, hemoglobin level, hypertension history, location of aneurysm, and Hunt-Hess grade were included in the propensity-score calculations. After 1:1 matching, 186 patients were identified in the normal and QTc prolongation groups, respectively. The baseline characteristics were confirmed to be equally distributed in both groups (Table 3). Before PSM, patients with QTc prolongation had higher mRS scores (worse outcome) ( 2.0 ± 2.0 vs. 1.2 ± 1.4, *p*<0.001 ) at 1 year follow-up. This result was confirmed after PSM [1.9 ± 1.9 vs. 1.6 ± 1.7, 95% CI 1.003 (0.999–1.008), *p*<0.001 ]. Of note, before PSM, patients with QTc prolongation had higher rate of perioperative cardiac arrest or ventricular arrhythmias ( 3.6% vs. 0.7%, *p* = 0.005 ). However, there was no statistical difference between normal and QTc prolongation groups in perioperative cardiac events after PSM (Table 4).

**Table 3:** Basic characteristics of aSAH patients after PSM

	Normal QTc (*n* = 186)	Long QTc (*n* = 186)	*P* value
Age (years), mean±SD	56.9 ± 9.8	57.9 ± 9.7	0.194
Hb	137.5 ± 18.0	135.7 ± 19.0	0.325
Hypertension	107 (57.5%)	108 (58.1%)	0.916
Location of aneurysm, n (%)			
Anterior circulation	167 (89.8%)	170 (91.4%)	0.594
Posterior circulation	19 (10.2%)	16 (8.6%)	
Hunt-Hess grade			0.472
I-III	171 (91.9%)	167 (89.8%)	
IV	15 (8.1%)	19 (10.2%)	

Abbreviations: aSAH, aneurysmal subarachnoid hemorrhage; Hb, hemoglobin; mRS, modified Rankin Scale; QTc, corrected QT interval

**Table 4:** Perioperative cardiac events and neurologic outcomes at 1 year follow-up before and after PSM in aSAH patients

	Before PSM			After PSM			
	Normal QTc (*n* = 714)	Long QTc (*n* = 194)	*P* value	Normal QTc (*n* = 186)	Long QTc (*n* = 186)	*P* value	OR (95% CI)
mRS	1.2 ± 1.4	2.0 ± 2.0	<0.001	1.6 ± 1.7	1.9 ± 1.9	<0.001	1.003 (0.999–1.008)
Cardiac arrest or ventricular arrhythmias	5 (0.7%)	7 (3.6%)	0.005	2 (1.1%)	4 (2.2%)	0.685	

Abbreviations: aSAH, aneurysmal subarachnoid hemorrhage; mRS, modified Rankin Scale; PSM, propensity-score matching; QTc, corrected QT interval

## Discussion

aSAH is a fatal neurosurgical emergency, which has high morbidity and mortality. Patients after aSAH may complicate with ECG abnormalities, including bradyarrhythmias, tachyarrhythmias, T-wave inversion, ST segment elevation or depression, and pathologic Q-wave, which might predispose the patients with or are predictors of higher morbidity and mortality [17, 18]. It had been reported that QTc prolongation was associated with a five-fold increase of cardiac arrest and VAs in liver transplantation [12]. Bradycardia, relative tachycardia, and non-specific ST and T wave abnormalities are strongly and independently associated with 3-month mortality after aSAH [13]. However, whether QTc prolongation is a predictor of cardiac and non-cardiac morbidity and mortality and the relationship between QTc prolongation and mid to long-term outcome after aSAH is insufficiently studied and remains controversial [13–15].

In this study, we found that female sex, hypokalemia, posterior circulation aneurysm, and high Hunt-Hess grade are associated with QTc prolongation after SAH. In primary analysis before PSM, higher age(OR = 1.022,95%CI 1.001–1.042), posterior circulation aneurysm (OR = 2.67, 95%CI 1.406–5.072), and high Hunt-Hess grade (more than III) (OR = 10.621, 95%CI 7.021–16.066) were identified to be associated with worse outcome at 1 year follow-up. QTc prolongation was not demonstrated to be associated with worse outcome during our initial analysis. To minimize the confounding bias, a PSM analysis was performed to compare the clinical outcomes between patients with normal QTc and QTc prolongation. We found that, after PSM, QTc prolongation was strongly associated with worse outcome in 1 year follow-up. At the same time, we found that there was no statistical relationship between QTc prolongation and perioperative cardiac arrest or ventricular arrhythmias.

The QTc represents cardiac repolarization during the cardiac action potential phase [19]. It had been reported that QTc prolongation was present in 40–70% of patients after SAH, which was 21.3% in our study [1, 6]. The inconsistency in QTc prolongation reported between studies may be due in part to differences in definitions of QTc prolongation. Studies have set different criteria for QTc abnormalities. QTc value of 440 ms is treated as a borderline for QTc prolongation, although this value is exceeded by approximately 10–20% of the general population [20]. The upper limits of normal QTc values are 470 ms in males and 480 ms in females. For both males and females, QTc ≥ 500 ms is considered highly abnormal [20, 21]. In our study, QTc ≥ 470ms in male and ≥ 480ms in female is considered as QTc prolongation.

QTc prolongation may provoke arrhythmias such as Tdp. Tdp is life-threatening since it easily develops into ventricular fibrillation and leading to sudden death. Though QTc prolongation has been reported to contribute to a five-fold increase in the risk of perioperative cardiac arrest and VAs in liver transplantation, in this study, we did not demonstrate a causal relationship between QTc prolongation and perioperative cardiac arrest or VAs in patients with aSAH [12].

The cause of QTc prolongation after SAH remains unclear. One of the most popular hypotheses is increased release of or enhanced sensitivity to catecholamines after SAH [17, 22, 23]. Following SAH, acute sympathetic surge causing an acute increase of circulating catecholamines or marked increase of catecholamines in myocardium even without a significant increase in plasma [17]. Animal study showed that hearts from animals following experimental SAH exhibit enhanced sensitivity to norepinephrine infusion and sympathetic stimulation [23].

Female sex and hypokalemia are independent risk factors for severe QTc prolongation in patients with SAH [3]. It has also been observed that the ECG of rats with hypokalemia showed QTc prolongation [24]. Our study is consistent with these results. In addition, we also demonstrated that older age, hypertension, posterior circulation aneurysm, and higher Hunt-Hess grade were also associated with QTc prolongation in patients following SAH. In univariate analysis, we found that QTc prolongation was strongly associated with worse neurologic outcome and perioperative cardiac arrest and ventricular arrhythmias. However, multivariate logistic analysis showed no statistical relationship between QTc prolongation and neurologic outcome. To further minimized the confounding bias, we performed PSM, which showed that patients with QTc prolongation had higher mRS scores (worse outcome) at 1 year follow-up. But there was no statistical difference between normal and QTc prolongation groups in perioperative cardiac events. Hence, we may deduce that QTc prolongation is associated with worse outcome in patients following SAH, which is independent of perioperative cardiac events.

We acknowledge there were some limitations in this study. Firstly, it is a retrospective study, some of the key information were missed. Most of the patients’ medical history was provided by their family members, and there was recall bias. In most of the cases, ECG data before aSAH were not available. Hence, the causal relationship between aSAH and QTc prolongation could not be strongly supported only by preoperative ECG after aSAH ictus. Secondly, follow-up ECG after microsurgical clipping or embolization of the responsible aneurysms were also not available in most of the cases, which hindered further analysis between aneurysm securing and ECG alteration. Hence, prospective studies with sufficient sample size and multiple-centers are anticipated. Thirdly, the SAH patients were experienced with aneurysm clipping or embolization, both the operators and operation time have an impact on the prognosis of patients, which was a cause of bias. Besides, patients with poor grade SAH are likely to have non neurological manifestations of other organs and cardiac issues including QTc prolongation. So, in these patients, poor grade SAH might be responsible for poor mRS and not mere QTc prolongation. QTc prolongation may be associated with but not for poor outcomes.

In conclusion, advanced age, posterior circulation aneurysm, Hunt-Hess grade more than III are associated with poor prognosis in patients with SAH. QTc prolongation is associated with poor prognosis following SAH, which still needs to be verified by further prospective clinical trials. QTc prolongation is not associated with perioperative CA/VA in this study.

## Data Availability

The datasets used and/or analyzed in the current study are available from the corresponding author on reasonable request.

## Abbreviations

aSAH: Aneurysmal subarachnoid hemorrhage
ECG: Electrocardiograph
PSM: Propensity score matching
QTc: Corrected QT interval
Td: Torsades de pointes
VAs: Ventricular arrhythmias
